# Dissecting Complex Traits Using Omics Data: A Review on the Linear Mixed Models and Their Application in GWAS

**DOI:** 10.3390/plants11233277

**Published:** 2022-11-28

**Authors:** Md. Alamin, Most. Humaira Sultana, Xiangyang Lou, Wenfei Jin, Haiming Xu

**Affiliations:** 1 Institute of Bioinformatics, Zhejiang University, Hangzhou 310058, China; 2 Department of Biology, School of Life Sciences, Southern University of Science and Technology, Shenzhen 518055, China; 3 Department of Biostatistics, University of Alabama at Birmingham, Birmingham, AL 35294, USA

**Keywords:** linear mixed model (LMM), GWAS, complex traits, omics, interaction effect

## Abstract

Genome-wide association study (GWAS) is the most popular approach to dissecting complex traits in plants, humans, and animals. Numerous methods and tools have been proposed to discover the causal variants for GWAS data analysis. Among them, linear mixed models (LMMs) are widely used statistical methods for regulating confounding factors, including population structure, resulting in increased computational proficiency and statistical power in GWAS studies. Recently more attention has been paid to pleiotropy, multi-trait, gene–gene interaction, gene–environment interaction, and multi-locus methods with the growing availability of large-scale GWAS data and relevant phenotype samples. In this review, we have demonstrated all possible LMMs-based methods available in the literature for GWAS. We briefly discuss the different LMM methods, software packages, and available open-source applications in GWAS. Then, we include the advantages and weaknesses of the LMMs in GWAS. Finally, we discuss the future perspective and conclusion. The present review paper would be helpful to the researchers for selecting appropriate LMM models and methods quickly for GWAS data analysis and would benefit the scientific society.

## 1. Introduction

Genome-wide association study (GWAS) is the most popular strategy for dissecting complex traits of agronomical significance and human diseases with the rising of cutting-edge microarray and next-generation sequencing (NGS) tools and the development of linear mixed models [1,2]. GWAS has been applied to many complex human traits, including diabetes, cancer, and several inflammatory diseases and detected hundreds of novel genes [3,4]. Many studies have also been effectively executed in plants, including the model plant Arabidopsis [5] and other plants [6,7,8,9,10]. Several factors contribute to this success, such as high-throughput technological advancement [11,12], the HapMap project [13], and the growth of advanced statistical methodologies for GWAS [14]. However, few genetic elements related to most traits have been identified that are explained by the genes in GWAS [15], which could be due to several reasons; for example, the influence of a single variant on a disease trait could be of imperfect penetrance and poor power to identify uncommon variants associated with disease, and epistatic and/or gene–environment (G × E) interactions [16]. Moreover, significant single nucleotide polymorphisms (SNPs) can only account for a small part of genetic contributions to complex traits or diseases [17]. 

The major problems in GWASs are confounding factors, including population stratification, familial correlation, and relatedness among individuals [18,19,20,21,22]. LMM (linear mixed model), also called mixed linear model (MLM), genomic control (GC), family-based association test, structured association, and principal components analysis are the statistical methods for correcting these confounders. LMMs can manage these confounders better, compared with other methods [22]. These methods proved useful in adjusting the inflation from many minor genetic outcomes and correcting the bias of population structure [19,23,24]. A combination of fixed and random effects is used to model phenotypes in the LMMs approaches, where the effect of candidate SNPs is considered fixed, and the random effects account for polygenic background variables with a covariance matrix across individuals [22]. LMMs are extensively applied in the genetic analysis of quantitative traits in plants and humans [25], which are attractive, familiar, and adaptable methods as they provide the individuals’ genetic effects in GWAS [25,26]. Previous studies showed that the mixed models could well accommodate population stratifications by calculating phenotypic covariance resulting from the genetic relatedness or relationship among individuals, and had functioned well in GWAS [18,20,21,27,28]. A study to investigate the epistatic and G × E interactions using an LMM showed that epistasis and G × E interactions are crucial components of the genetic architecture of complex diseases [29].

Recently, several papers have been published to highlight the importance of LMMs in GWAS [26,30,31]. These studies concentrated on a specific topic using LMMs in different fields. However, no studies have covered all of the currently available LMMs methods on GWAS in the literature. Therefore, we aim to provide a thorough review on available LMM methods for GWAS. First, we discuss diverse LMM approaches, including single locus, multi-locus, multivariate/multi-traits, epistasis (G × G) and gene–environment (G × E) interaction, TWAS (transcriptome-wide association studies), and longitudinal GWAS. Then we present different packages and web-based software/server tools using LMMs. Moreover, we have discussed the advantages and weaknesses of the linear mixed models utilized in GWAS. Finally, we discuss the future perspective and conclusion of the present study. Existing publications were collected in PubMed, Google Scholar, Web of Sciences, and other search engines, including Bing. Publications not associated with LMMs applied in GWAS were excluded in the present review. 

## 2. Linear Mixed Models 

**Figure 1:**
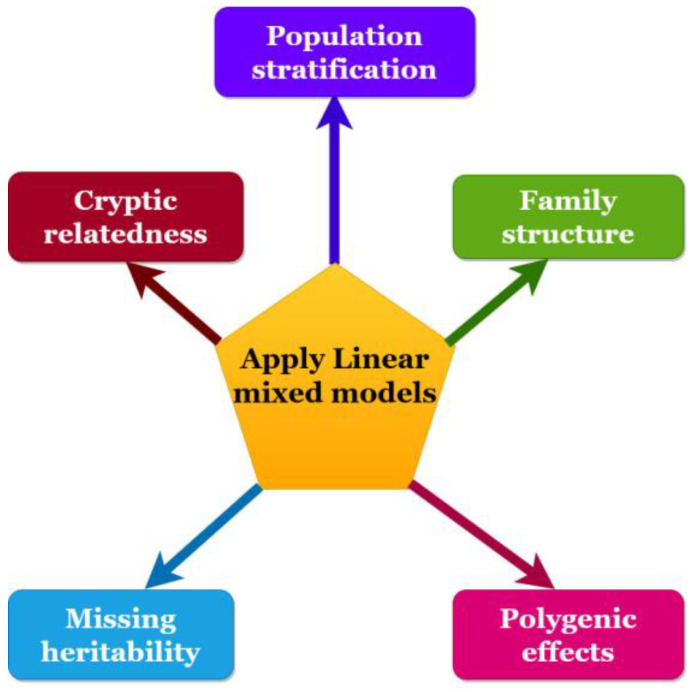
Problems solved by LMMs in GWAS for dissecting complex traits.

LMMs can solve different problems, including population stratification, family structure, cryptic relatedness, estimating polygenic effect, and missing heritability (Figure 1). 

**Figure 2:**
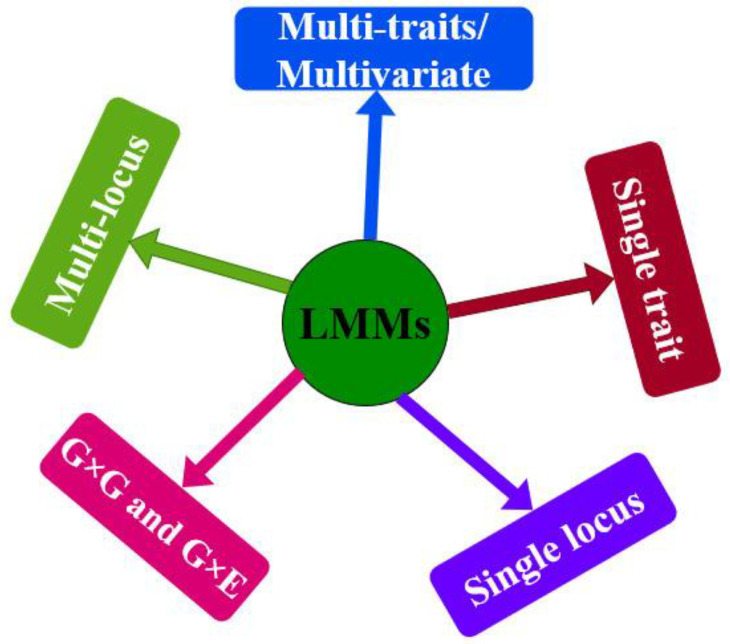
Types of LMMs used in GWAS for dissecting complex traits.

The LMMs were originated to account for multiple levels of relatedness by using a kinship matrix, which greatly enhanced the performance of GWAS by reducing both the false-positive and the false-negative rates [18]. LMMs increase the power to discover QTNs (quantitative trait nucleotides) by governing the false-positive rates in presence of confounding factors, including population structure and cryptic relatedness [18]. Different types of LMMs, including single locus, multi-locus, multi-traits/multivariate, gene by gene (G × G) and gene by environment (G × E), have been used in GWAS for dissecting complex traits (Figure 2). We briefly discuss each type of LMM in the following subsections.

### 2.1. LMMs for Single Locus Analysis 

Many LMM methods have been proposed and applied in GWAS according to the recent advancement [19,20,21,23,27,32] since the first study [18,33]. A single-locus LMM for the n measurement of a phenotype across l inbred strains can be written as the previous study defined [19] as follows:

(1) y=Xβ+Zu+e

where *y* is an n×1 dimensional observed phenotype, X is an n×q dimensional fixed effect matrix with mean, SNPs, and different confounding variables. β is a q×1 dimensional fixed effect coefficient parameter. Z is an n×l incidence matrix mapping every observed phenotype to one of the l inbred strains; u is the random effect with $Var\;\left(u\right)=\sigma_{u}^{2}K,$ where K is the l×l kinship matrix, and e is an n dimensional residual effect such that $Var\;\left(e\right)=\sigma_{e}^{2}I$. The respective paper can find details about parameter estimation and polygenic background controlling strategy for each single-locus model.

Studies confirmed that the methods of controlling the population structure and the confounding factors had better performance than those that did not consider confounding factors [20,21]. For example, EMMA (efficient mixed-model association), an LMM model, is used for adjusting genetic relatedness and population structure in GWAS [19]. EMMA showed more effectiveness than the classical LMM method, which used spectral decomposition to change the calculation process. EMMAX (EMMA eXpedited), a variance component approach, decreased the computational time for analyzing big GWAS data sets [21]. CMLM (compressed MLM) and P3D (population parameter previously determined) remove the re-calculation of variance components, resulting in significantly decreased computational time and improved statistical power [20]. CMLM substitutes the individuals’ genetic impact with the clusters of similar individuals based on their association obtained from entirely obtainable genetic markers [20]. Statistical power was enhanced by 5–15% compared to the conventional LMM method, and computational time decreased using the CMLM method. FaST-LMM (factored spectrally transformed linear mixed models) used the subset of markers to manage the polygenic effect, resulting in accelerated speed and needing less memory [27]. It expressively improved computational speed by using a rank-reduced kinship algorithm, which depends on a subset of fewer genetic markers than the number of individuals [34]. GRAMMAR, genome-wide rapid association using a mixed model and regression, calculates the residuals at the beginning and then dissects the association utilizing LMM [32]. 

RMLM (random-SNP-effect MLM) considers the SNP-effect as random and permits using Bonferroni correction to estimate the *p*-value for significance tests [24]. The identified markers are concurrently assessed in a single model employing an EM empirical Bayes approach in the next phase of GWAS [35]. ECMLM (enriched CMLM) allows researchers to select numerous algorithms to cluster individuals into groups and several measurements to originate group kinship from single kinship, resulting in increased statistical power in GWAS for complex traits [36]. FaST-LMM-Select is a simple empirical method that gives enhanced power and adjustment [37]. First, it ranks the SNPs from the lowest to the highest based on the *p*-values obtained by linear regression, then constructs a genetic similarity matrix involving SNPs until it detects the first minimum in the GC factor (λGC). The GRAMMAR-Gamma method has been proposed as an analytical estimate within the basis of the score test technique [38]. This method gives unbiased estimates of the SNP effect and has power approximate to the LRT-based method, and it can be used for a large human cohort in GWAS. The computational burden of this method is near its theoretical minimum, and the running time is linearly related to the sample size [38]. 

SUPER (Settlement of MLM Under Progressively Exclusive Relationship) extracted a small subgroup of SNPs and applied them in FaST-LMM [39]. SUPER follows several steps. In the first step, the whole genome is which split into small bins, where the best important marker presents each bin. Subsequently, it selects only the influential bins and applies an ML (maximum likelihood) method to improve the size and the number of bins taken as the possible QTNs causal of the phenotypes. Finally, the small set of markers is used to define the kinship among the individuals by omitting the markers in LD (linkage disequilibrium) for testing the marker irrespective of local distance [39]. SUPER is computationally fast and outstandingly gains statistical power despite utilizing the whole set of SNPs [39]. WarpedLMM (warped linear mixed model) method simplifies the ordinary LMM that estimates an ideal conversion from the monitored data for genetic study [25]. It can also be adjusted for more particular tasks, such as for analysis of multi-locus or multiple phenotypes, and results demonstrated that transformations derived from WarpedLMM enhanced power and accuracy in GWAS. Recently, GMMAT (generalized linear mixed model association test) has been proposed, which is computationally useful for analyzing binary traits using a logistic mixed model approach for GWAS [40]. GMMAT applied a mixed logistic model once per GWAS and executed score tests under the null hypothesis, and it successfully controlled population structure and relatedness when examining binary traits in various study designs [40]. LMM-Score (LMM employing the score test) is a new method proposed to identify the genetic loci of complex traits [1]. This method employs a score test that does not need to estimate parameters under the full model. This method has increased power and requires less computing time than the traditional LMM method in calculating trait heritability. For interested users and readers to select the best method among the single-locus LMM models, the authors suggest top models in sequential order, which are based on the maximum number of citations in Google Scholar given below:

EMMAX > EMMA > CMLM/P3D > Fast-LMM > GRAMMAR > GMMAT > RMLM > GRAMMER-Gamma. These orders are based on the most cited to less cited models, and this style is followed for all other cases to suggest the models that may be chosen by the researchers in this manuscript. The single-locus model and their respective software and packages for GWAS using LMM are given in Table 1. 

### 2.2. LMMs for Multilocus Analysis

Most of the methods perform a single-dimensional genome scan by testing one single marker at a time, where multiple test adjustments are needed for the cut-off value of the significance test. Several single-locus methods such as EMMAX [19], P3D [20], FaST-LMM [27], and GEMMA [23] have been suggested to facilitate the computational load. Most quantitative traits are regulated by a few genes with significant effects and many polygenes with small effects [26]. However, most studies have utilized single-locus GWAS methods, including LMM models and limited algorithms applied to multi-locus GWAS [41]. 

Multi-locus methods consider all loci info together and do not need multiple test corrections due to the nature of multi-locus [24]. Some multi-locus methods, including MLMM, MRMLM, FASTmrEMMA, FASTmrMLM, and FarmCPU using LMM, have been proposed and demonstrate more statistical power than single locus methods [24,26,42,43,44]. 

A multi-locus model can be written as the extended version of Equation (1) followed by the previous study, defined [24,43] as follows:

(2) $$y=X\beta +\sum_{k}Z_{k}u_{k}+\xi +e$$

where $Z_{k}$ is a vector of genotype indicators for the kth SNP, $u_{k}$ is the effect of marker k and $u_{k}\sim \;N\left(0,\;\sigma_{k}^{2}\right)$, $\xi \;\sim \;MVN\left(0,\;K\sigma_{g}^{2}\right)$ is a vector of polygenic effect with a multivariate normal distribution with mean zero and variance $\sigma_{g}^{2}$ described by the kinship matrix K, $e\;\sim \;MVN\left(0,\;I\sigma_{e}^{2}\right)$ is the residual error with an identity matrix $I_{n\times n}$, and other notations are the same as in Equation (1). In the respective papers can be found details about parameter estimation and polygenic background controlling strategy for each multi-locus model. For example, a multi-locus model named MLMM (multi-locus mixed-model) used forward inclusion and backward exclusion in selecting loci [42]. Results showed that MLMM performs better than the existing methods concerning power and FDR (false discovery rate) for analyzing GWAS data with complex traits [42]. LMM-Lasso aggregates multi-variable association analysis with perfect improvement for population structure [45]. It permits jointly detecting various loci with minor effects while considering potential structure between samples [45]. It is theoretically easy, computationally effective, and balances genome-wide settings. PUMA (Penalized Unified Multiple-locus Association), utilizing a family of GWAS data, consists of a class of statistical procedures developed to discover poor associations that are not predicted by conventional analytical approaches [46]. It can handle thousands of genetic markers in a single statistical model by employing the penalized ML structure utilizing a generalized linear model. Results showed that PUMA had improved power to identify weak associations compared to usual GWAS and former penalized methods [46].

**Table 1:** Single-locus model and their respective software and packages for GWAS using LMM.

Tool	Description	Link	Effect			Polygenic Background			Reference
			*a*	*d*	*α*	*a*	*d*	*α*	
GRAMMAR	GRAMMAR is an alternate method to pedigree-founded QTL association mapping, which is quick and easy. It can handle millions of markers and is significantly faster than the evaluated genotype approach for association analysis.				✓			✓	[32]
EMMA	EMMA is a fixed model edition of LMM used to control GWAS’s population structure and genetic relatedness.	http://mouse.cs.ucla.edu/emma/ (accessed on 20 October 2022)			✓			✓	[19]
CMLM/P3D	CMLM (compressed MLM) diminished the sample size into groups using the clustering method, P3D (population parameters previously determined), which removes the re-calculation of variance components. The combined application of these two methods prominently abridged computing time and retained/enhanced statistical power.	https://www.maizegenetics.net/tassel (accessed on 20 October 2022)			✓			✓	[20]
EMMAX	EMMAX is a variance component approach founded on the LMM method, which decreases the computational time for analysis of big GWAS data sets and is used for fixing sample structure in GWASs.	http://genetics.cs.ucla.edu/emmax/ (accessed on 20 October 2022)	✓						[21]
FaST-LMM	FaST-LMM, an LMM-based method, used the subset of markers to manage the polygenic effect, resulting in accelerated speed and less required memory for GWAS.	https://github.com/fastlmm/FaST-LMM/ (accessed on 20 October 2022)			✓			✓	[27]
FaST-LMM-Select	FaST-LMM-Select is a simple method that shows that wisely choosing a reduced number of SNPs consistently enhances power, expands standardization, and decreases computational time.	http://mscompbio.codeplex.com/ (accessed on 20 October 2022)			✓			✓	[37]
GRAMMAR-Gamma	GRAMMAR-Gamma is an exceptionally fast variance component-based method that can be used for the massive human cohort in GWAS. It is established based on the analytical approximation within the context of the score test method.	http://www.genabel.org/ (accessed on 20 October 2022)			✓			✓	[38]
WarpedLMM	WarpedLMM is a simplification of the ordinary LMM that estimates an ideal transformation from the monitored data for genetic study. Subsequently, this method’s power and accuracy will increase in GWAS.	http://github.com/pmbio/warpedLMM (accessed on 20 October 2022)			✓			✓	[25]
ECMLM	ECMLM, enriched CMLM, uses various related algorithms and then selects the most effective mixture between the relationship algorithm and grouping algorithm resulting in increased power and can be applied for complex traits.	http://www.maizegenetics.net/gapit (accessed on 20 October 2022)			✓			✓	[36]
SUPER	SUPER method intensely decreases the number of genetic markers utilized to define individual relationships, resulting in fast computation and increased statistical power despite utilizing the whole set of SNPs.	http://www.zzlab.net/GAPIT/ (accessed on 20 October 2022)			✓			✓	[39]
RMLM	RMLM, random-SNP-effect MLM, treats the SNP-effect as random and uses Bonferroni correction to determine the *p*-value for significance.				✓			✓	[24]
GMMAT	GMMAT is an R package for carrying out association tests using GLMMs in GWAS and sequencing association studies.	https://cran.r-project.org/web/packages/GMMAT/index.html (accessed on 20 October 2022)			✓				[40]
LMM-Score	LMM-Score is a new method proposed to identify the genetic loci of complex traits. The simulation study showed that this method’s power increased and needed less computing time than the traditional LMM methods.				✓			✓	[1]

Note: *a*: additive effect; *d*: dominant effect; *α*: allelic substitution effect, *α* = *a* + *d*(*q* − *p*), where *p* and *q* are the frequencies of alleles *A* and *a*, respectively. The effect and polygenic background in all tables were partially adopted from another study, described elsewhere [47].

mrMLM (multi-locus RMLM) uses markers selected from the RMLM method with a flexible selection criterion, and is more reliable in QTN discovery and more precise in the QTN effect estimation than the RMLM and EMMA [24]. Recently, FASTmrEMMA, a fast multi-locus random-SNP-effect EMMA, has been proposed to improve the existing multi-locus GWAS method [26]. It used the MLM and EMEB (expectation and maximization empirical Bayes) methods together, where marker effects were considered random, and then the multi-locus model was applied to utilize the EMEB method [26]. The results showed that FASTmrEMMA is more reliable in QTN identification, has a smaller bias in QTN effect calculation, and needs less computation time than current methods, including SUPER, EMMA, CMLM, and ECMLM. FarmCPU (Fixed and random model Circulating Probability Unification) model is proposed to remove confounding factors and is currently frequently used in GWAS [44]. The power of this method increases, along with control of the false-positive rate and needs reduced calculation times compared with existing methods [44]. FASTmrMLM is a more robust method using the previously suggested mrMLM [24] integrated with GEMMA and matrix transformation [43]. More than 50% of computational time decreased, statistical power improved in QTN discovery, and reduced false positive rate by FASTmrMLM instead of GEMMA, MRMLM, and FarmCPU [43]. 

StepLMM (stepwise LMM) is a consistent, versatile, and computationally proficient method that can be applied to both GS (genomic selection) and GWAS [48]. It used LMMs and a kinship matrix to control the population stratification, and the variance components were re-calculated by an efficient mixed method at each regression stage. StepLMM used the Bayes information criteria as convergence conditions, and valuable and rigorous measures for model assessment in GWAS [49]. A new multi-marker method called SGL-LMM was recently proposed, which combined SGL (sparse group lasso) and LMM to control confounding factors in GWAS [50]. Results showed that the SGL-LMM improved its power to detect marker association in many settings and is suitable for GWAS [50]. For interested users and readers to select the best method among the multi-locus LMM models, the authors suggest top models in the sequential order based on the maximum number of citations in Google Scholar as follows:

BOLT-LMM > MLMM > FarmCPU > mrMLM > FASTmrEMMA > LMM-Lasso. Researchers could use these methods based on their research interests or data types. The multi-locus models and their respective software and packages for GWAS using LMM are given in Table 2.

**Table 2:** Multi-locus models and their respective software and packages for GWAS using LMM.

Tool	Description	Link	Effect			Polygenic Background			Reference
			*a*	*d*	*α*	*a*	*d*	*α*	
MLMM	MLMM, a multi-locus mixed-model, is an LMM-based method for complex traits, which is computationally effective and shows excellent performance regarding power and FDR compared with existing methods.	https://github.com/Gregor-Mendel-Institute/mlmm (accessed on 20 October 2022)	✓	✓		✓	✓		[42]
LMM-Lasso	LMM-Lasso links the benefits of LMM with Lasso regression, which is free of tuning parameters and efficiently corrects population structure. LMM-Lasso instantaneously detects potential causal variants and provides multi-marker-founded phenotype prediction from genotype.	https://github.com/BorgwardtLab/LMM-Lasso (accessed on 20 October 2022)	✓			✓			[45]
Puma	PUMA, a standard model for utilizing a family of GWAS data, has been proposed to detect a weak association that the traditional methods cannot identify. It used a penalized maximum likelihood method utilizing a general linear model to take thousands of markers in a particular statistical method instantaneously.	http://mezeylab.cb.bscb.cornell.edu/Software.aspx (accessed on 20 October 2022)			✓			✓	[46]
BOLT-LMM	BOLT-LMM is an efficient LMM that is computationally fast and gains power by demonstrating more accurate, non-infinitesimal genetic designs through a Bayesian admixture preceding marker impact.	http://www.hsph.harvard.edu/alkes-price/software/ (accessed on 20 October 2022)			✓			✓	[51]
mrMLM	mrMLM (multi-locus RMLM) used markers selected from the RMLM method with a flexible selection criterion, and simulation results showed that the mrMLM is stronger in QTN discovery and more precise in QTN effect estimation than the RMLM and EMM.	https://cran.r-project.org/web/packages/mrMLM/index.html (accessed on 20 October 2022)	✓			✓			[24]
*FarmCPU*	*FarmCPU was* formulated *to control the confounding factors, significantly enhance* statistical power, and decrease computing power.	https://www.zzlab.net/FarmCPU/ (accessed on 20 October 2022)			✓			✓	[44]
FASTmrEMMA	FASTmrEMMA, a dominant multi-locus model widely used in QTN identification and model fit, has a lower bias in QTN effect calculation and needs a lower running time than existing single- and multi-locus methods.	https://cran.r-project.org/web/packages/mrMLM/index.html (accessed on 20 October 2022)			✓			✓	[26]
StepLMM	StepLMM is a consistent, versatile, and computationally proficient method that can be applied to GS and GWAS. StepLMM has excellent efficiency in both GWAS and GS and is workable for agronomic breeding and human genomic studies.				✓			✓	[48]
FASTmrMLM	FASTmrMLM is a multi-locus method, which is a fast and authentic algorithm in GWAS and assures superior statistical power, high accuracy of estimates, and low false-positive rate.	https://cran.r-project.org/web/packages/mrMLM/index.html (accessed on 20 October 2022)	✓			✓			[43]
SGL-LMM	SGL-LMM, a multi-marker method, combined SGL and LMM for controlling confounding factors in GWAS. It includes the effect of multiple markers and integrates biological group info as preceding evidence in the model.				✓			✓	[50]

Note: *a*: additive effect; *d*: dominant effect; *α*: allelic substitution effect, *α* = *a* + *d*(*q* − *p*), where *p* and *q* are the frequencies of alleles *A* and *a*, respectively.

### 2.3. Multivariate/Multi-Traits LMMs

Multivariate LMMs are commonly used to assess the association between SNPs and multiple correlated phenotypes in genetics due to their effectiveness in controlling relatedness amongst samples [23]. Many multi-trait models have been used for a prolonged period in quantitative genetics [52,53,54,55], but these approaches have hardly been used for GWAS. Multivariate LMMs are widely used in the different fields of genetics, such as the identification of QTL [56], evaluating the pleiotropy and genetic association amongst complex phenotypes [57,58,59] and realizing evolutionary forms [60]. These models are widely acceptable in GWAS not only for their application in sample relatedness and governing population stratification but also for their admiration of the power of the possible advance from multivariate GWAS [53,54,57,61,62,63] compared with univariate [18,19,20,21,23,27,64,65,66]. A multivariate/multi-traits model to analyze associations between the ith SNP and the jth phenotype can be written as follows:

(3) $$\;y_{j}=X_{i}\beta_{j}+u_{j}+e_{j},$$

where $y_{j}$ is a vector of length n with the jth phenotype, $X_{i}$ is a vector of length n with genotypes of the ith SNP, $\beta_{j}$ is the effect of the ith SNP on the jth phenotype, $u_{j}$ contains the effect of population structure of the ith SNP on the jth phenotype, and $e_{j}$ is the residual error of the *j*th phenotype. According to the single-locus LMM model in Equation (1), each phenotype follows a multivariate normal distribution with mean $X_{i}\beta_{j}$ and variance $\sum_{j}$, where $\sum_{j}=\sigma_{g_{j}}^{2}K$ **+** $\sigma_{e_{j}}^{2}I$ is the variance of the *j*th phenotype. The details about the multi-traits model and variance-covariance matrix calculation can be found elsewhere [67].

Korte, Vilhjalmsson [57] initially used multivariate LMMs for pairwise quantitative trait analysis in a human cohort. They proposed MTMM (multi-trait mixed mode) for associated phenotypes considering both between- and within-trait variance components concurrently for multiple traits for adjusting population stratification in GWAS [57]. MTMM performed better than single-trait LMMs in identifying loci and could also break down overall trait covariance into genetic and environmental factors. Fitting multivariate LMMs needs a computationally demanding parameter estimation process, where their application has been bound to two traits till now [57,67,68]. GEMMA (genome-wide efficient mixed-model association) has been proposed for fitting multivariate LMMs, which enhances power and computational speed more than the previous methods such as GCTA [69] and WOMBAT [70] and can include more than two phenotypes in the model [23]. It fits BSLMM for effectively integrating the benefits of both LMMs and sparse regression, is robust to different settings in the proportion of variance in phenotypes explained (PVE) estimation, and outperforms in phenotype prediction. Moreover, it can handle three types of models, such as univariate and multivariate LMM and Bayesian sparse LMM. GEMMA can adapt a reasonable number ranging from 2–10 phenotypes and demonstrates computation considerably quicker than MTMM. mvLMM (matrix-variate linear mixed model), a further advanced method, needs less computational time to execute ML inference in a multi-trait model using data transformation [67]. Human data analysis proved that mvLMM increased computational speed ten times, resulting in a practically used large population in GWAS [67]. 

However, while various multivariate methods are proposed to discover variants linked to more than one phenotype, these existing approaches do not investigate the population structure [71]. GAMMA (generalized analysis of molecular variance for mixed-model analysis) considered the population structure in the model, which can instantly analyze multiple phenotypes and adjust population structure [71]. Results indicated that GAMMA is an enhancement over former methods [19,72] that can detect accurate signals or generate numerous false positives. The existing methods apply a particular area to improve the required computations in multi-traits mixed model approaches. LIMIX is a simple and effective LMM-based software with concurrence to Python for multi-traits genetic analysis [73]. It permits the demonstration of genomic or environmental elements by aggregating diverse fixed effects. It can easily adjust mixed models for various uses with diverse observed and secret covariates and flexible study purposes. Results showed that LIMIX enhances power and prediction precision, particularly while incorporating stepwise multi-locus regression into multi-trait models and examining huge numbers of traits [73]. WOMBAT is software used for the quantitative genetic study of continuous multi-traits using REML (restricted maximum likelihood) [70]. It permits various models, fitting several traits, fixed and random effects, designated genetic covariance configurations, and abridged rank approximation. WOMBAT is well-fitted to investigate big GWAS data sets, assuring both computational effectiveness and authentic maximization of the likelihood function [70]. 

Methods for assessing a set of variants are crucial for GWAS with complex traits [74]. Set tests are a regression model used for essaying statistical dependencies amongst sets of genetic variants and an objective quantitative trait. This test can be attained by applying LMM through an accumulation of additive effects of multiple variants in a particular variance component [75]. Set tests can help abridge the amount of genome-wide tests and are efficient when the causative variant is unseen or when many causal variants are present compared with single-variant methods [76]. However, the current set test did not account for confounding factors, which is a central problem for the big genomic data set to increase statistical power. FaST-LMM-Set for set tests has been proposed to handle confounding problems based on the LMM and used two random effects [74]. It used the LRT (likelihood ratio test) and score test, and the results showed that LRT gives more power to controlling type-I error. A second random effect has recently been included in the set tests to control confounding factors, heritable background effects, and relatedness [74,77,78]. A useful set test named mtSet (multi-trait set test) has been proposed for joint analysis throughout numerous linked traits when considering population structure and relatedness and can be applied to one and several traits in GWAS [75]. mtSet is based on a multivariate LMM with two variance components and is computationally capable and facilitating genetic analysis for large cohorts [75]. SMMAT (set mixed-model association test) is a computationally effective variant set test for continuous and binary traits [79]. It can be used in structured and related samples with various possible correlations from large-scale whole-genome sequencing studies. It is supposed that SMMAT could help better understand the complex traits and diseases in human genetic investigation with the technological advances and analytical approaches in large-scale GWAS [79]. 

Using the variance component (VC model), LRT-based VC studies [18,19,27] are the standard of genetic association. VC studies have gained attention for analysis of human complex traits and application in various fields, including inheritable phenotypic variation elucidated by SNPs [69,80], its allocation across chromosomes, allele frequencies, and functional annotations [81], and its connection all over traits [58]. Though LRT-based VC methods need to estimate all model parameters for each tested genetic marker, existing VCMs such as GCTA [69] become computationally intensive when the population sizes are over 50,000. To overcome this problem, a two-stage approach was suggested instead of the ordinary LRT [64]. The two-step approach would estimate the LRT quickly, if many loci of minor effects participated in trait finding [21,64], and be computationally faster than the LRT-based approach. The BOLT-RELM method is a much faster VC method and can handle large samples [82]. It uses the Monte Carlo average information REML algorithm [83], which approximates Newton-type maximization of the restricted log-likelihood concerning the calculated variance parameters [82]. GCTA and BOLT-REML used REML to estimate genetic correlation amongst two traits of any kind, whereas the mvLMM method is close to GEMMA and can solely adapt normally distributed traits [82]. Although all three approaches apply similar algorithms, BOLT-REML and mvLMM are more effective than GCTA concerning run time and memory utilization [67,82]. Another efficient LMM method, BOLT-LMM, needs just a few O(MN)-time repetitions and gains power by demonstrating more accurate, non-infinitesimal genetic designs through a Bayesian admixture preceding marker impact [51]. Results revealed that cohort size power gains allow BOLT-LMM to favor big cohorts’ data in GWAS. Penalized-MTMM combines both the within- and between-trait variance factors for multiple traits [84]. This method uses AI-REML to calculate variance components and deals with variable selection by applying group MCP (minimax concave penalization) and point estimation using sparse group MCP [84]. LiMMBo (linear mixed models with bootstrapping) has been proposed to facilitate the computationally efficient combined genetic study of multi-dimensional phenotypes [85]. It cuts the number of operative model parameters by entering a mediate subsampling step, strongly controlling the population structure. It can be used for handling big GWAS data with hundreds of traits. All multi-trait LMM methods are popular. For interested users and readers to select the best method among the multivariate/multi-traits LMM models, the authors suggest top models in the sequential order based on the maximum number of citations in Google Scholar as follows:

GEMMA > WOMBAT > BOLT-REML > MTMM > LIMIX. The widely used method’s recommendation could help the users and readers make a quick decision and save time in analyzing their GWAS data using a multivariate/multi-traits LMM model. Multi-trait/multivariate model and their respective software and packages for GWAS using LMM are given in Table 3.

### 2.4. Linear Mixed Models in Epistasis (G × G) and Gene-Environment (G × E) Interaction

Though many optimistic results have been produced using the different methods in GWAS data analysis, it has been recognized that additive effects can elucidate only a portion of genetic variations [86]. Epistasis is considered a reasonable basis for undetermined variations [87,88]. Much research in epistatic interactions has been completed for complex human traits [89], suggesting that more research about interactions among genetic variants is uncovered. Many software such as INTERSNP [90], EpiGPU [91], FastEpistasis [92], EPIBLASTER [93], TEAM [94], and methods [88,95] have been proposed considering the interaction between two loci for big omics datasets. An epistasis (G × G) and gene–environment (G × E) interaction model for mapping the SNPs in the homozygote population and transcripts/proteins/metabolites in homozygote/heterozygote population for the *k*-th subject and *h*-th environment can be written by the following LMM [96]:

(4) $$y_{kh}=\;\mu +e_{h}+\underset{i}{\sum }c_{i}u_{ik}++\underset{i<j}{\sum }cc_{ij}u_{ijk}+\underset{i}{\sum }ce_{ih}u_{ikh}+\underset{i<j}{\sum }cce_{ijh}u_{ijkh}+\varepsilon_{kh}$$

where *μ* is the population mean; $e_{h}\;$ is the fixed effect of the *h*-th ethnic population; $c_{i}$ is the *i*-th locus effect with coefficient $u_{ik}\;(1\;\mathrm{for}\;QQ,-1\;\mathrm{for}\;qq\;\mathrm{and}\;0\;\mathrm{for}\;Qq$ in QTS mapping, and expression values using in QTT/P/M mapping); $cc_{ij}$ is the epistasis effect of locus i× locus j with coefficients $u_{ijk}\;(1\;\mathrm{for}\;QQ\times QQ\;\mathrm{and}\;qq\times qq,-1\;\mathrm{for}\;QQ\times qq\;\mathrm{and}\;qq\times QQ\;$ in QTS mapping, and expression values $u_{ik}\times u_{jk}\;$ using in QTT/P/M mapping); $ce_{ih\;}$ is the environment interaction effect of the *i*-th locus and the *h*-th environment with coefficient $u_{ikh}$; $cce_{ijh}$ is the epistasis × environment interaction effect of locus i× locus j in the *h*-th environment with coefficient $u_{ijkh}$; $\varepsilon_{kh}$ is the residual effect of the *k*-th individual in the *h*-th environment. The details about parameter estimation and test statistic for *G* × *G* and *G* × *E* interaction models can be found elsewhere [96].

**Table 3:** Multi-trait/multivariate models and their respective software and packages using LMM.

Tool	Description	Link	Effect						Polygenic Background						Reference
			*a*	*d*	*α*	*ae*	*de*	*αe*	*a*	*d*	*α*	*ae*	*de*	*αe*	
WOMBAT	WOMBAT is a software package that analyzes multiple quantitative traits using REML. It is well-fitted to investigate big GWAS data sets and assure both computational effectiveness and accurate boosting of the likelihood function.	http://didgeridoo.une.edu.au/km/wombat.php (accessed on 21 October 2022)	✓			✓									[70]
GEMMA	GEMMA (genome-wide efficient mixed-model association) is used to calculate precise values of test statistics and is constructed on EMMA software. It can handle three types of models such as univariate and multivariate LMM and Bayesian sparse LMM.	http://www.xzlab.org/software.html (accessed on 21 October 2022)			✓						✓				[23]
MTMM	MTMM is an LMM method for associated phenotypes considering both between and within-trait variance components concurrently for multiple traits for adjusting population stratification in GWAS.	https://github.com/arthurkorte/MTMM (accessed on 21 October 2022)			✓			✓						✓	[57]
FaST-LMM-Set	FaST-LMM-Set, a novel approach for set tests, can handle the confounding problem. It is based on the LMM and uses two random effects: the first random effect is used to capture the set association signal, and the second is used to control confounding factors.	http://mscompbio.codeplex.com (accessed on 21 October 2022)			✓						✓				[74]
mtSet	Set tests are an effective approach for genome-wide association essaying among groups of genetic variants and a single quantitative trait. mtSet is an application of effective set test algorithms for combined analysis across multiple traits, which can explain confounding factors, including relatedness and single and multiple traits that can be used for GWAS.	https://github.com/PMBio/mtSet (accessed on 21 October 2022)			✓						✓				[75]
LIMIX	LIMIX, a simple and effective LMM-based software, can execute a wide range of genetic analyses for multi-trait using GWAS data. It can handle diverse functions, including single-locus and interaction association studies and variance decomposition studies with LMMs.	https://limix.readthedocs.io/en/s/ (accessed on 21 October 2022)			✓			✓						✓	[73]
BOLT-REML	BOLT-REML uses the RELM approach to estimate the variance parameters for models, taking multiple variance components and traits that solve computational problems that make it impossible to analyze large data sets.	https://www.hsph.harvard.edu/alkes-price/software/ (accessed on 21 October 2022)			✓						✓				[82]
mvLMM	mvLMM (matrix-variate linear mixed model) is a multiple-trait association mapping approach, which needs less computational time to execute inference in a multi-trait model by using data transformation and a ten-fold computational speed increase for large cohort analysis.	http://genetics.cs.ucla.edu/mvLMM (accessed on 21 October 2022)			✓						✓				[67]
GAMMA	GAMMA, a multivariate method, can coincidentally analyze numerous phenotypes and adjust for population structure. GAMMA is a more advanced method than others, which either cannot find true effects or have a higher false positive rate.	http://genetics.cs.ucla.edu/GAMMA/ (accessed on 21 October 2022)			✓						✓				[71]
LiMMBo	LiMMBo is a very easy and flexible method based on LMMs for multi-dimensional GWAS data with hundreds of phenotypes. It combines LMMs and bootstrapping for estimates of large trait covariance matrices.	https://github.com/HannahVMeyer/limmbo (accessed on 21 October 2022)			✓						✓				[85]
SGL-LMM	SGL-LMM combined SGL (sparse group lasso) and LMM for multivariate GWAS analysis. Results showed that the SGL-LMM improved the power to detect marker association in various settings.				✓						✓				[50]
SMMAT	SMMAT is a computationally effective variant test for continuous and binary traits. SMMAT can be used in structured and related samples with various possible origins of correlations from large-scale whole-genome sequencing studies.	https://github.com/hanchenphd/GMMAT (accessed on 21 October 2022)			✓						✓				[79]

Note: *a*: additive effect; *d*: dominant effect; *α*: allelic substitution effect, *α* = *a* + *d*(*q* − *p*), where *p* and *q* are the frequencies of alleles *A* and *a*, respectively; e: environmental effect; *ae*: additive-environment interaction effect; *aa* (*aae*): additive-additive epistatic effect (or interaction effect between *aa* and environment); *ad*: additive-dominant effect; *da*: dominant-additive effect; *dd*: dominant-dominant effect.

FAM-MDR, a multifactor dimensionality reduction technique, detects epistasis in minor or extensive pedigrees [97]. It aggregates characteristics of GRAMMAR with model-based multifactor dimensionality reduction. This model can manage complex and significant pedigrees with extra unconnected individuals [97]. The FAM-MDR methodology comprises two parts, where residuals are inferred from a polygenic model. Both additive polygenic and confounding effects are removed at the first step. In the second step, FAM-MDR used a model-based MDR method for calculating the association between the new traits (residuals inferred in the first part are regarded as the new traits in the subsequent part of the FAM-MDR) and genotypes obtained based on the multi-locus dimensions [98]. The *p*-values for the best model can be estimated after randomly permuting the traits under the assumption of familial correlation-free traits in this step [97]. Simulation and real data analysis results showed that the FAM-MDR method performs better for solving multiple-testing problems, improves power, and expeditiously applies the whole available information compared with PGMDR [97]. Zhang, Zhu, Tong, Zhu, Qi, and Zhu [96] developed an association analysis method that can analyze epistasis (G × G) and G × E (genotype-by-environment) interaction based on a mixed linear model. However, it cannot be directly used for high-density SNP marker data, and many markers need to be screened before analysis. They implemented their method in software named QTXNetwork, based on the graphics processing unit system to analyze diverse genetic effects concurrently. Three functional modules, including QTL identification, QTS (quantitative trait SNP) detection and QTT/P/M (quantitative trait transcript/protein/metabolite) analysis, can be done using QTXNetwork. Simulation study and real data analysis proved that unbiased estimation would be found for genetic effects by QTXNetwork [96]. 

A study showed that the LMMs were unable to control the inflation of test statistics for G × E but were only capable of handling population structure when considering the genetic relatedness in the model [99]. To overcome this problem, the researcher considered traditional genetic similarity and the associated individuals with identical environments, which causes misleading G × E interactions [99]. Another method named iSet was proposed based on LMMs, considering G × E in the model and answering for polygenic effects [98]. This study showed that the model’s power increased due to considering the interactions with variants; consequently, this method detected many unknown interactions [98]. Research showed that epistasis allows a practicable path for investigating possible genetic systems of complex traits. However, computational efficiency is a great barrier to identifying interactions effect in real-world problems, particularly in controlling the type I error, population structure and cryptic relatedness using the LMMs [100]. REMMA, a rapid epistatic mixed-model association, has been proposed to address these issues based on the knowledge of approximation between GBLUP (genomic best linear unbiased prediction) and SNP-BLUP [100]. This model has several advantages, such as computational efficiency, lower Type I error rate, and QTL discovery power [100]. However, the computational complexity is O(n2), where n is the population size. Therefore, the same group proposed the REMMAX (REMMA eXpedited) model to reduce the computational time for the epistatic GWAS model. REMMAX can concurrently manage association studies for additive × additive, additive × dominance, dominance × dominance, and individual-definite residual effects for controlling background by integrating various polygenic effects in the model [97]. Additionally, the fairly accurate REMMAX algorithm suggested filtering out the non-significant interactions and then applying a Wald $\chi^{2}$ test to accelerate the computation times. Accordingly, time complexity reduced and became linear with the population size, and real data analysis results revealed that REMMAX is a proficient method for interpreting genetic structures of complex traits [101].

G × E interaction can detect the genetic effects, which are avoided in the linear models, enhance the GWAS power, and give the fractional answer to the missing heritability [102,103]. Another group proposed GxEMM, an integrative mixed model for polygenic interactions, to obtain the total effect of small G × E effects to disseminate throughout the genome [104]. Most importantly, environmental variables are not necessarily categorical, and diverse quantities of heritability could be assigned to diverse environments [105]. It can be employed for any GWAS datasets with pertinent environmental interaction and is especially useful when splitting heritability into distinct environmental components. For estimating G × E based-heritability, GxEMM elucidates key biases in the latest methods. For example, GxEMM can adapt to the overall environment, noise diversity, and binary traits [104]. 

Various phenotypes and environmental variables such as nutrition, physical exertion, or lifestyle covariates can help the G × E interaction study [103]. The study proved that phenotypes controlled by a single locus interacted with multiple environments. However, there are no powerful approaches for the joint G × E interaction study of multiple environmental variables. StructLMM (Structured LMM) has been proposed to analyze G × E interactions, which is computationally effective in detecting the loci that relate to hundreds of environmental variables [103]. This method possesses more power and enhances robustness in case of large numbers of environmental variables analysis compared with the conventional G × E interaction fixed effect test for single and manifold degrees of freedom [103]. Moreover, allelic effect size estimations, which contributed to G × E interaction, for each individual were obtained by this method. Recently, the deep mixed model has been proposed for random model interactions between SNPs for adjusting confounding factors in GWAS [106]. Grid-LMM is a scalable algorithm for frequently suiting complex LMMs that can include various origins of heterogeneity, including additive and dominance genetic variance and G × E interactions [107]. It is applied to execute the G × E interaction and find the association for phenotypes determined by a non-additive inherited variation, an advantage from prototyping multiple random effects [107]. Simulation and real data analysis results showed that accuracy for association investigation and power to discover causal genetic variants increased by Grid-LMM in GWAS [107]. It is a user-friendly method for genome-wide data that prominently decreases their computational load, and users can easily select the best statistical model for analyzing their data [107]. FFselect is an LMMs-based advanced method for analyzing GWAS data incorporating shared environmental effects in the model [108]. Phenotypic variance can be subdivided into large, small, and environmental genetic effects, which permits the user to estimate the environmental variance by FFselect [108]. Additionally, this method supplies an understanding of trait genetic structure founded on the many loci with larger genetic effects. Furthermore, this method incorporated auxiliary criteria to stop the forward feature assortment of pseudo QTNs to avoid overfitting problems [108]. This method demonstrated enhanced power, effectively controlled FDR, and simultaneously adapted for environmental factors to enlarge the effectiveness of GWAS. A study evaluated the overall G × E interaction using LMMs [109]. Authors considered instantaneous scoring of particular and general environmental effects for fixed effect terms demonstrating G × E effects in this study. The genomic inflation factor is controlled by considering both G × E and G × T (genotype by trial) effect for random effects terms [109]. The LMM approach was applied to tomato phenotype data collected in two different seasons. Results showed that this method identified both QTLs with consistent effects throughout the cultivating seasons and G × E effects. Moreover, this study discovered more QTLs with G × E effects than other LMM methods [109]. Recently, Li, et al. [110] established a compressed variance component mixed model framework, namely 3VmrMLM (three-variance-component mixed model), to detect QTNs and QTN × E and QTN × QTN interaction and estimate all their possible effects by controlling all the possibly polygenic backgrounds. Simulation and real data analysis showed that 3VmrMLM has more power, accuracy, and a small FDR [110]. Moreover, this model has the facility to handle compound environments to discover QTN × E interaction and variable selection beneath a polygenic setting for finding QTN × QTN interaction [110]. Many G × G and G × E interaction LMM methods have been proposed but the results obtained by different methods across environments are not stable. Researchers can use the newly developed method 3VmrMLM, which considered all possible interactions and controlled all possible polygenic backgrounds, which might provide better results. Additionally, the relevant software named IIIVmrMLM [47] can easily be used for the analysis of GWAS data. G × G and G × E interaction and their respective software and packages for GWAS using LMM are given in Table 4. 

### 2.5. Linear Mixed Models in Transcriptome-Wide Association Studies (TWAS) and Longitudinal GWAS

**Figure 3:**
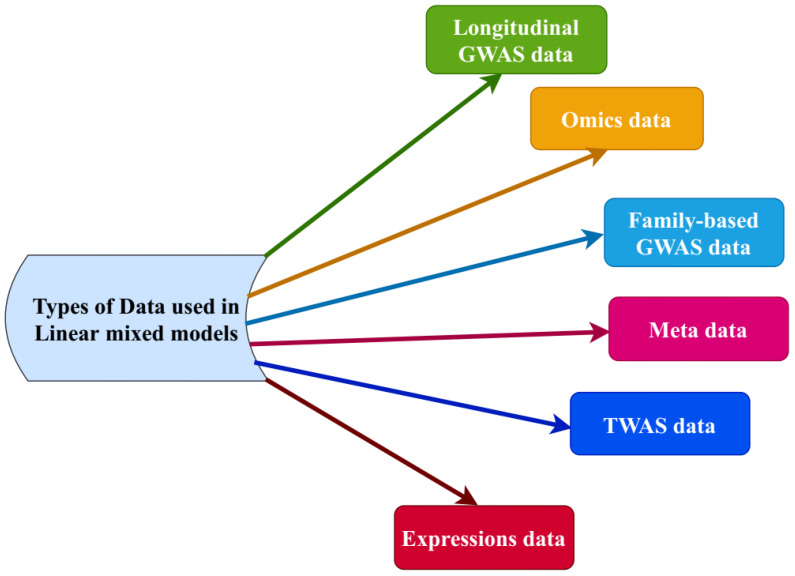
Different types of data can be analyzed by LMMs in GWAS for dissecting complex traits.

GWAS has been effectively used for discovering various genetic variants linked with complex traits/diseases [111]. However, the mechanism behind the genetic variants linked to the complex traits is unclear [111]. Different types of data, including Omics-, clustered-, longitudinal-, family-based GWAS-, expression-, TWAS-, and meta-data, can be handled by LMMs (Figure 3). Recent studies assume genetic variants regulate complex traits by affecting cellular traits, including protein overflow and gene expression [112,113]. LSMM (latent sparse mixed model) method incorporates genetic and cell-type functional annotations with GWAS data [114]. It uses the EM algorithm for parameter estimations and statistical inference. Results showed that the LSMM has more power than current methods in detecting the risk variants (SNPs) and cell-type targeted functional observations and consequently brings about insightful knowledge of the genetic architecture of complex traits in GWAS [114]. 

SMART (Scalable Multiple Annotation integration for trait-Relevant Tissue identification) is based on the extension of LMM [115]. This model assumes that all SNP effects follow a random distribution. SMART integrates numerous SNP operative annotations from omics investigations on GWAS summary data to assist the detection of trait-associated tissues to reconstruct the dominant association test [115]. CoMM (collaborative mixed model) has been proposed to investigate the mechanism related to linked variants in complex traits [111]. CoMM is computationally fast and statistically effective in analyzing genetic contributions to complex traits by maximizing information in transcriptome data.

**Table 4:** Epistasis (G × G) and gene–environment (G × E) interaction and their respective software and packages for GWAS using LMM.

Tool	Description	Link	Effect									Polygenic Background								Reference
			*a*	*d*	*α*	*e*	*aa/aae/ae*	*ad/* *ade*	*da/* *dae* */de*	*dd/dde*	*qqe*	*a*	*d*	*α*	*e*	*aa/* *ae*	*ad*	*da/* *de*	*dd*	
FAM-MDR	FAM-MDR, a novel family-based and compromising epistasis finding exploration method, provides better results than the existing method PGMDR (Pedigree-based Generalized MDR) in terms of power, and it sufficiently contracts with numerous testing in epistasis tests.	http://www.statgen.be/ (accessed on 21 October 2022)			✓									✓						[97]
QTXNetwork	QTXNetwork is an LMM-based software that uses GPU to analyze diverse genetic effects concurrently. It can be used for calculating main genetic effects, *G × G* and *G × E* interaction effects on big omics data for complex traits and for calculating the heritability of specific genetic component effects.	http://ibi.zju.edu.cn/software/QTXNetwork (accessed on 21 October 2022)	✓	✓	✓	✓	✓/✓	✓/✓	✓/✓	✓/✓	✓									[96]
iSet	The interaction set test, iSet, is an LMMs-based method that explains the polygenic effects and has more power to detect the interaction between environment and variants.	https://github.com/limix/limix (accessed on 21 October 2022)				✓								✓						[98]
REMMA	REMMA has been proposed to overcome the computational efficiency problem for handling epistatic effects in GWAS. It is more computationally efficient, has a lower type I error rate, and has higher QTL discovery power than other existing models.	https://github.com/chaoning/REMMA (accessed on 21 October 2022)	✓				✓													[100]
GxEMM	GxEMM is an integrative mixed model for polygenic interactions to disseminate the total effect of small G × E effects throughout the genome.	https://github.com/andywdahl/gxemm (accessed on 21 October 2022)			✓		✕/✓									✕/✓				[104]
StructLMM	StructLMM (structured linear mixed model) is a computationally effective method to detect and illustrate loci that relate to one or more environments. Hundreds of environmental variables can be used to study interactions using this model.	https://mybinder.org/v2/gh/limix/limix-tutorials/master?filepath=struct-lmm.ipynb (accessed on 21 October 2022)			✓		✕/✓							✓						[103]
Grid-LMM	Grid-LMM is a scalable algorithm for frequently suiting complex LMMs that can include heterogeneity, including additive and dominance genetic variance, uneven distribution of traits, and G × E interactions.	https://github.com/deruncie/GridLMM (accessed on 21 October 2022)			✓		✕/✓													[107]
FFselect	FFselect is an LMM based advanced method for the analysis of GWAS data incorporating shared environmental effects in the model. This method demonstrated enhanced power, controlled FDR (false discovery rate), and simultaneously adapted to environmental factors to enhance GWAS’s effectiveness.	https://github.com/NicholSchultz/FFselect (accessed on 21 October 2022)			✓	✓														[108]
REMMAX	REMMAX, REMMA eXpedited, is a proficient method for GWAS by adjusting numerous polygenic effects, and the time complexity is almost linear with the population size.	https://github.com/chaoning/GMAT (accessed on 21 October 2022)	✓	✓			✓	✓		✓		Polygenic background with normal distribution								[101]
3VmrMLM	3VmrMLM, a three-variance-component mixed model, was incorporated with the mrMLM method. It has more power and accuracy to discover all kinds of loci and give an unbiased estimation of their effects.		✓	✓		✓	✓/✕ /✓	✓/✕	✓/✕/✓	✓/✕		✓	✓		✓	✓/✓	✓	✓/✓	✓	[110]

Note: *a*: additive effect; *d*: dominant effect; *α*: allelic substitution effect, *α* = *a* + *d*(*q* − *p*), where *p* and *q* are the frequencies of alleles *A* and *a*, respectively; e: environmental effect; *aa/aae/ae*: additive-additive epistatic/interaction effect between *aa* and environment/additive-environment interaction effect; *ad/ade*: additive-dominant effect/interaction effect between *ad* and environment; *da/dae/de*: dominant-additive effect/interaction effect between *da* and environment/dominant-environment interaction effect; *dd/dde*: dominant-dominant effect/interaction effect between *dd* and environment; *qqe*: interaction effect between *qq* and environment.

Real data analysis demonstrated that CoMM could identify more genetically governed genes associated with complex traits deprived of excessive type I errors. However, CoMM is an effective method, but it uses individual-level GWAS data and cannot entirely use extensively existing summary statistics data in GWAS [116]. CoMM-S^2^ methods proposed using summary statistics GWAS data rather than individual-level data [117]. This method uses similar approaches to CoMM except for summary statistics data. CoMM-S^2^ has some benefits over CoMM. For example, CoMM-S^2^ is computationally more proficient than CoMM when using larger sample sizes. The authors showed that CoMM-S^2^ performed better when the cellular heritability was small [116]. However, CoMM-S^2^ cannot be applied in a cross-tissue study. Additionally, CoMM-S^2^ cannot differentiate whether the discovered genes are only correlated with the complex traits or if they are genuine causal effects [116]. 

Numerous GWASs have been implemented in population cohorts that have repeated measures at multiple time points for each individual [117,118,119], but usual association methods only take account of one-time points. Furlotte et al. [120] offered a mixed-model-based longitudinal GWAS, which used multiple phenotype measurements for every individual. Their model clarifies phenotypic chronological tendencies and uses a kinship coefficient matrix-based LMEM (linear mixed-effects model) named KIN-LMEM to control population structure. The results demonstrated that power was improved compared with conventional methods [120]. Additionally, it is feasible to separate the genetic effect from the environmental effect when the manifold measurement for a unique individual is accessible using the KIN-LMEM method. Even if this method essayed for a specific set of assumptions, it may also be utilized for a larger class of challenges [120]. Another method based on a conditional two-step approach was proposed for longitudinal data, suggesting a computationally realistic result for inquiring about the association between the provided SNP and the longitudinal desire trait [121]. Sikorska et al. [122] applied a quick conditional two-step method founded on fitting an LMEM accompanied by linear regression as a computationally efficient solution for LMEM with random intercept and slope. Sung et al. [123] proposed two-stage approaches for family-based data to detect the pleiotropic impact on multiple longitudinal traits. Among the TWAS and longitudinal LMM methods, KIN-LMEM is a very popular and widely used method. We suggest choosing the TWAS models from the sequential order based on the maximum number of citations in Google Scholar as follows: KIN-LMEM > SMART > CoMM > CoMM-S^2^ > LSMM. TWAS and longitudinal-related LMM models, software, and packages for GWAS are given in Table 5.

### 2.6. LMM-Based Packages in GWAS

Many LMM-based packages have been developed in GWAS (Table 6). DMU is a broadly employed package in quantitative genetics and is applied to estimate the variance components, fixed effects, and predict random effects [124]. This package analyzes MMM (multivariate mixed models) under continuous improvement for over 30 years. Many high-performance methods have been applied for particular project-associated tasks and common applications in genetics and genomics research-related packages integrated with the DMP package [124]. GenABEL is another widely used software R library for GWAS, which is very useful for verifying the quality of genetic data, screening associations between SNPs with binary or quantitative traits, displaying results, and delivering comfortable interfaces to ordinary statistical results and figures [125]. lrgpr is a high-functioning and convenient R interface for assessing LMMs [126]. Lrgpr has been configured for interactive and big-scale GWAS analysis for the confounding effects of family relationships and population stratification.

**Table 5:** Transcriptome-wide association studies (TWAS) and longitudinal-related LMMS models, software, and packages for GWAS.

Tool	Description	Link	Effect				Polygenic Background				Reference
			*a*	*d*	*α*	e	*a*	*d*	*α*	e	
SMART	SMART is based on the extension of LMM that utilizes various corresponding annotations matched to diverse approaches and algorithms. SMART can be applied to construct useful SNP set experiments and decide novel trait-tissue related and useful annotations concerning trait-tissue associations.	http://www.xzlab.org/software.html (accessed on 21 October 2022)			✓				✓		[115]
LSMM	LSMM incorporates both genic and cell-type targeted functional annotations in GWAS. It uses the EM algorithm for parameter estimations and statistical implications. The power increased compared with current methods to detect the risk variants (SNPs) and cell-type targeted functional observations by the LSMM approach.	https://github.com/mingjingsi/LSMM (accessed on 21 October 2022)			✓				✓		[114]
CoMM	CoMM, a collaborative mixed model, is to inquire about the recurring role of linked variants in complex traits. CoMM is computationally fast and statistically effective in analyzing genetic contributions to complex traits by maximizing information in transcriptome data.	https://github.com/gordonliu810822/CoMM (accessed on 21 October 2022)			✓				✓		[111]
CoMM-S^2^	CoMM-S^2^ uses summary statistics GWAS data to study the mechanism of genetic variants. This method uses similar approaches to CoMM, except for summary statistics data and simulation and real data analysis showed that the efficiency of CoMM-S^2^ is equivalent to CoMM and CoMM-S^2^ applied in the CoMM package.	https://github.com/gordonliu810822/CoMM (accessed on 21 October 2022)			✓				✓		[116]
KIN-LMEM	KIN-LMEM is a mixed-model-based approach for executing association mapping, which utilizes numerous phenotype measurements for each individual.	http://genetics.cs.ucla.edu/longGWAS/ (accessed on 21 October 2022)			✓	✓					[120]

Note: *a*: additive effect; *d*: dominant effect; *α*: allelic substitution effect, *α* = *a* + *d*(*q* − *p*), where *p* and *q* are the frequencies of alleles *A* and *a*, respectively; e: environmental effect.

**Table 6:** LMM-based packages in GWAS.

Tool	Description	Link	Effect			Polygenic Background			Reference
			*a (aa/ad)*	*d* (*dd*/*da*)	*α*	*a*	*d*	*α*	
DMU	DMU is a broadly employed package for analyzing MMM in quantitative genetics and genomics. It applies advanced tools to calculate variance components and fixed effects and predict random effects.	http://dmu.agrsci.dk (accessed on 21 October 2022)	✓						[124]
ASREML	ASReml utilizes LMMs to analyze big and complex data, and many variance models for random effects are available in the LMM in the ASReml package.	https://www.vsni.co.uk/ (accessed on 21 October 2022)			✓			✓	[127]
GenABEL	GenABEL is an R package GWAS, which applies an efficient GWA data storehouse and dealing, quick processes for verifying the quality of genetic data, statistical analysis, and representation of GWAS data.	https://mran.microsoft.com/snapshot/2018-05-12/web/packages/GenABEL/index.html (accessed on 21 October 2022)							[125]
lrgpr	lrgpr is very computationally powerful and efficient for analyzing big GWAS and NGS datasets. It provides a collaborative model conforming to assist exploratory data analysis from the perspective of the LMM.	http://lrgpr.r-forge.r-project.org/ (accessed on 21 October 2022)			✓			✓	[126]
lme4qtl	lme4qtl, an extension of lme4, adds novel models for genetic studies and extends a flexible model for settings with numerous levels of connection and would be useful while covariance matrices are sparse.	https://github.com/variani/lme4qtl (accessed on 21 October 2022)			✓			✓	[128]
Sci-LMM	SciLMM is a systematic model for analyzing the ancestries of millions of individuals. SciLMM uses LMM approaches in the presence of the dependencies encoded by matrices constructed by the model. This tool is adaptable, can be elongated in various ways, and is valuable for GWAS.	https://github.com/TalShor/SciLMM (accessed on 21 October 2022)	✓ (✓/✓)	✓ (✓/✓)					[129]
Single-RunKing	Single-RunKing is a useful R package to speed up the computation in GWAS by using LMMs. It uses R/fastLmPure to numerically understand the genetic effects of screened SNPs and concentrate on significant SNPs found by the EMMAX algorithm.	https://rdrr.io/cran/RcppBlaze/man/fastLmPure.html (accessed on 21 October 2022)			✓			✓	[130]
LiMMBo	LiMMBo is a very easy and flexible method based on LMMs for multi-dimensional GWAS data with hundreds of phenotypes. It combines LMMs and bootstrapping for estimates of large trait covariance matrices.	https://github.com/HannahVMeyer/limmbo (accessed on 21 October 2022)			✓			✓	[85]
SGL-LMM	SGL-LMM combined SGL (sparse group lasso) and LMM for multivariate GWAS analysis, with improved power to detect marker association in various settings.	https://rdrr.io/cran/RcppBlaze/man/fastLmPure.html (accessed on 21 October 2022)			✓			✓	[50]
SMMAT	SMMAT is a computationally effective variant test for continuous and binary traits. SMMAT can be used in structured and related samples with various possible origins of correlations from large-scale whole-genome sequencing studies.	https://rdrr.io/github/hanchenphd/GMMAT/man/SMMAT.html (accessed on 21 October 2022)			✓			✓	[79]

Note: *a* (*aa/ad*): additive effect (or additive-additive epistatic effect or additive-dominant effect); *d*: dominant effect (or dominant–dominant effect or dominant–additive effect); *α*: allelic substitution effect, *α* = *a* + *d*(*q* − *p*), where *p* and *q* are the frequencies of alleles *A* and *a*, respectively.

Linear and logistic regression models can be fit using this software, which permits accommodating millions of regression models on a desktop by employing an effective execution, concurrent, and out-of-core data processing for big datasets. ASReml, a statistical package, utilizes LMMs by REML for big datasets with complex variance frameworks [127]. Many variance models for random effects are available in the LMM in the ASReml package. Another package named lme4qtl, an extension of lme4, is the most effective method for QTL mapping [128]. It proposes a flexible model for settings with numerous levels of kinship and becomes efficient while covariance matrices are sparse. Family-based data were used to show that lme4qtl is a computationally effective and useful tool. 

Single-RunKing, an efficient R software, has been proposed to speed up the GWAS by LMMs [130]. It uses R/fastLmPure to numerically understand the genetic effects of screened SNPs and concentrate on significant SNPs found by the EMMAX algorithm. LMMs and their annexes have currently acquired significant acceptance in human genetics research for estimating heritability [69,80,83,131,132,133], genetic correlation [58,134], predicting phenotype [66,135,136,137], and design sample kinship [22,51,138]. Nevertheless, LMMs have not yet been utilized to study population-scale human genealogies. Shor, Kalka, Geiger, Erlich, and Weissbrod [129] proposed Sci-LMM (Sparse Cholesky factorization LMM), a systematic model for analyzing ancestries with millions of individuals. Sci-LMM can build a matrix of relationships among trillions of pairs of people and fit the representing LMM in a few hours. It offers an integrated basis for inquiring about the epidemiological record of human populations through a pedigree track record and is useful for GWAS [129]. For interested users and readers to select the best method among the LMM-based packages in GWAS, the authors suggest top packages in the sequential order based on the maximum number of citations in Google Scholar as follows: ASREML > GenABEL > DMU > lme4qtl > SMMATs. Shortly, ASREML is broadly employed for big and complex GWAS data, and GenABEL is well-known for inspecting the quality and demonstration of the GWAS data. Moreover, DMU is usually used for calculating variance components and fixed effects and predicting the random effect, lme4qtl is appropriate for the sparse covariance matrix, and SMMATs are mostly used for continuous and binary traits. Every package has different types of advantages and disadvantages. Most curious researchers may check other packages and their details in Table 6.

### 2.7. Web-Based Software/Server Tools Using Linear Mixed Models

Many software and server-based tools have been developed for multi-omics data analysis in GWAS. Qxpak is a software-based mixed-model, which allows a versatile tool for QTL mapping in various populations, including cross-between inbred lines and within-population analysis [139]. Association studies between SNP and an interesting trait can be done using Qxpak. The most computationally demanding work for every SNP in succession throughout the genome is to fit an LMM, which is guided to improve numerous quicker estimations for building tests of the fixed SNP outcomes in the LMM [20,21,32,38,64]. These approximate tests have been used in various packages such as GenABEL [125], EMMAX [21], TASSEL (Trait Analysis by aSSociation, Evolution and Linkage) [140], and MMM [65]. TASSEL is a widely used software that applies a standard linear model and LMM methodologies for controlling population stratification and family architecture [140]. Traits association, evolutionary pattern, LD, and principal components analysis can be estimated using TASSEL. 

QTLNetwork is a widely used software for linkage mapping and visualizing the genetic structure for complex traits, where analytical populations are derived from a crossing of different inbred lines [141]. It can accommodate QTLs with special effects, epistasis, and Q × E (QTL-environment interaction) effect. QTLNetwork provides a GUI facility and can deal with data from diverse forms of observational populations. Although thousands of SNPs associated with complex traits have been identified using GWAS [142], only a portion of the heritability explained by the identified genome-wide significant SNPs due to the numerous SNPs with minor effects are still to be identified [17]. GCTA, genome-wide complex trait analysis, is a flexible tool to calculate and dissect complex trait variation using big GWAS data sets [69]. This method was developed to tackle the “missing heritability” problem. GCTA calculates the variance accounted for by all the SNPs on a chromosome for complex traits instead of testing the association of a single specific SNP to the trait. To investigate and enhance the knowledge about the genetic architecture of complex traits, GCTA covers many other analyses now [69]. GAPIT (Genome Association and Prediction Integrated Tool) applies innovative statistical procedures, including the CMLM (compressed mixed linear model) and CMLM-founded genomic prediction [143]. The GAPIT software offers multiple options for the necessary association tests and uses the most computationally effective methods, including MLM, CMLM, ECMLM, FaST-LMM, FaST-LMM-Select, and SUPER methods in the improved version of the GAPIT [34]. Recently, various powerful LMMs, including FaST-LMM-Select [28], ECMLM [36], and SUPER [39] have been implemented in the GAPIT version 2 [34]. The modified version is relatively easy to run and allows for journal-set-up tabular sum-ups and figures. 

MASTOR (mixed-model association score test on related individuals) has been proposed for genetic association mapping a quantitative attribute [144]. It can handle samples with linked individuals and attains high power by using full kinship information to integrate partly missing data in the investigation when adjusting for dependence [144]. Another widely used package is named MMM, which utilizes LMM with one random effect, whose covariance design can be easily assigned by the users for GWAS [65]. It can handle more than 20,000 individuals and 500,000 genetic variants and can be used with other types of data. MMM and FaST-LMM packages have been implemented in the GEMMA package, and those methods used the exact model increasing power relying on the true fundamental layer of relatedness [23,65]. OmicABEL considers the problem of mixed model-based GWAS for a random number of traits [145]. Results showed that different computational algorithms are best for analyzing single- and multi-trait mixed model-founded GWAS, and OmicABEL attains significant speed-ups compared with existing methods.

PEPIS (Pipeline for estimating EPIStatic) has been proposed to estimate polygenic effects based on the LMM [146]. PEPIS used C/C++ programming and integrated respective beneficial publicly available mathematical functions and upgraded libraries, which will tackle the existing problems in epistasis analysis in GWAS [146]. MTG2 is based on the LMM approach using GWAS data for analyzing complex traits [131]. MTG2 incorporated the average information algorithm and eigen decomposition of the genomic relationship matrix, which is considerably faster than other REML methods [131]. It could be applied for the highest number of statistical models than GEMMA, including MLMMs, random regression models, and numerous variance components approach. It can be a valuable and resourceful tool for complex traits studies, especially for multivariate analysis, such as estimating genetic variance-covariance and G × E. PopPAnTe, a versatile and straightforward software, has been proposed for pairwise association studies in associated samples with a wide range of predictors and response. It uses an exact LMM corresponding to that applied in the QTDT software [147]. It is very convenient for biobank data, where a wide range of pedigree evidence is missing [145]. GREML is a dominant LMM-based method where all SNP’s effects are collectively equipped as random effects and have been used for many traits, including height [80]. However, the GREML and Bayesian MLM methods did not examine the relationship between effect size and MAF (minor allele frequency) for complex traits. Bayesian LMM method has been proposed, named BayesS, which can concurrently estimate the effect size, MAF, SNP-based heritability, and polygenicity in usually unconnected individuals utilizing GWAS data [148]. BayesS is applied in a software tool called GCTB (genome-wide complex trait Bayesian analyses), and recently summary-data-based Bayesian LMMs integrated with the GCTB Version 2.0. 

OSCA was proposed to manage omics data from high-throughput trials in big cohorts and help analyze complex traits utilizing omics data [149]. OSCA used MLM-based omics association and multi-component MLM-based omics association, excluding the target method to discover omics associated with complex traits considering unseen confounding components and calculate the fraction of phenotypic variation caught by all quantities of one or different omics profiles [150]. Recently, an LMM-based computationally fast and efficient method, fastGWAS, was proposed to analyze biobank data [150]. This method was robust, authentic, and resource-effective for monitoring false positives in the presence of confounding factors, which is employed in the GCTA software package [150]. For interested users and readers to select the best web-based software/server tools and tools using LMM in GWAS, authors suggest top web-based software/server tools in the sequential order based on the maximum number of citations in Google Scholar as follows: TASSEL > GCTA > GAPIT > MMM > QTLNetwork > GAPIT Version 2 > GCTB > fastGWA > QxPak > fastGWA. These tools are very popular and widely used, and various association mapping can be done using these tools for analyzing complex traits based on the LMM model for GWAS. An interested user could investigate the other LMM-based software and server tools in more detail which are given in Table 7.

**Table 7:** Web software and server-based tools using LMMs.

Tool	Description	Link	Effect									Polygenic Background				Reference
			*a*	*d*	*α*	e	*ae*	*aa (aae)*	*ad*	*da*	*dd*	*a*	*d*	*α*	*e*	
QxPak	Qxpak is a mixed-model-based software that allows a very versatile tool for QTL mapping in various populations and can be used for multi-trait and multiQTL analysis in genomic studies.		✓	✓												[139]
TASSEL	TASSEL is software that measures trait associations, evolutionary patterns, and LD calculation. Database browsing and importing are assisted by incorporated middleware.	https://www.maizegenetics.net/tassel (accessed on 21 October 2022)			✓									✓		[140]
QTLNetwork	QTLNetwork is software for mapping and displaying the genetic structure underlying complex traits for observational populations that came from a cross relating to dual inbred lines. QTLNetwork provides a GUI facility and can deal with data from diverse forms of observational populations.	http://ibi.zju.edu.cn/software/qtlnetwork (accessed on 21 October 2022)	✓			✓	✓	✓ (✓)								[141]
GCTA	GCTA, genome-wide complex trait analysis, is a widely used software incorporating many methods for analyzing complex traits using GWAS.	https://cnsgenomics.com/software/gcta/ (accessed on 21 October 2022)				✓								✓		[69]
GAPIT	GAPIT applies promoted statistical approaches, including the CMLM and CMLM-based CMLM-founded genomic prediction.	https://www.maizegenetics.net/GAPIT (accessed on 21 October 2022)	Several methods including EMMA, P3D/CMLM, ECMLM, MLMM, SUPER and FarmCPU implemented in GAPIT. See the effect and polygenic background in the respective methods tables.													[143]
MASTOR	MASTOR is a mixed model-based approach for analyzing GWAS data using the score test for genetic association with a quantitative trait, where sample individuals are related. MASTOR attains high power by using full kinship information to integrate partly missing data in the investigation when adjusting for dependence.	http://www.stat.uchicago.edu/%7Emcpeek/software/MASTOR/index.html (accessed on 21 October 2022)			✓									✓		[144]
MMM	MMM, a software package, used LMM with one random effect whose covariance design can be easily assigned by the users for GWAS. It can handle more than 20,000 individuals and 500,000 genetic variants and use other data.				✓									✓		[65]
OmicABEL	OmicABEL is freely accessible software that carries out fast mixed-model-based GWAS. It can handle single and multi-trait and uses CLAK-C HOL to explore significant complex traits, and CLAK-E IG is used for investigating the genomic control of various omics in GWAS.	http://www.genabel.org/packages/OmicABEL (accessed on 21 October 2022)			✓											[151]
GAPIT Version 2	GAPIT version 2 included some powerful LMMs, including FaST-LMM-Select, ECMLM, and SUPER.	https://www.zzlab.net/GAPIT/ (accessed on 21 October 2022)	GAPIT version 2 is an updated version of GAPIT. Several methods including FaST-LMM and FaST-LMM-Select along with others methods mentioned in the GAPIT implemented in GAPIT version 2. See the effect and polygenic background in the respective methods tables.													[34]
PEPIS	PEPIS is a web-based tool for studying polygenic epistatic effects founded on an LMM employed to predict the functioning of hybrid rice. PEPIS was devotedly formulated to calculate epistatic effects and will help tackle the obstacles in genetic epistasis study.	http://bioinfo.noble.org/PolyGenic_QTL/ (accessed on 21 October 2022)	✓	✓				✓(✕)	✓	✓						[146]
MTG2	MTG2 is an LMM-based software for analyzing complex traits using GWAS data. It incorporated AI algorithms and eigendecomposition, which is considerably faster than other REML methods.	https://sites.google.com/site/honglee0707/mtg2 (accessed on 21 October 2022)	✓		✓											[131]
PopPAnTe	PopPAnTe, an easy Java program based on the accurate LMM, allows a flexible permutation method to end the propagation of arbitrarily permuted samples. It could be used for the exact relationship between significant quantitative response and independent variables in family-based GWAS data.	https://sites.google.com/site/populationgenomics/poppante (accessed on 21 October 2022)			✓									✓		[145]
GCTB	GCTB is a software tool that includes a class of Bayesian LMMs for complex trait studies applying genome-wide SNPs for dissecting complex traits. It offers users many functions to reveal necessary signatures of evolution.	https://cnsgenomics.com/software/gctb/ (accessed on 21 October 2022)			✓											[148]
OSCA	OSCA, a multipurpose software tool, manages omic data produced from high-throughput trials in big cohorts and helps analyze complex traits utilizing omic data.	https://cnsgenomics.com/software/osca/ (accessed on 21 October 2022)			✓											[149]
fastGWA	fastGWA, an LMM model, is proposed for controlling population structure by PCA and relatedness by sparse GRM (genetic relationship matrix) for analyzing big data such as biobank-scale data in GWAS.	http://cnsgenomics.com/software/gcta/#fastGWA (accessed on 21 October 2022)			✓									✓		[150]

Note: *a*: additive effect; *d*: dominant effect; *α*: allelic substitution effect, *α* = *a* + *d*(*q* − *p*), where *p* and *q* are the frequencies of alleles *A* and *a*, respectively; e: environmental effect; *ae*: additive-environment interaction effect; *aa* (*aae*): additive-additive epistatic effect (or interaction effect between *aa* and environment); *ad*: additive-dominant effect; *da*: dominant-additive effect; *dd*: dominant-dominant effect.

## 3. Advantages and Weaknesses of Linear Mixed Models Used in GWAS

LMMs are attractive because they can control population structure and explain polygenic information for typical single-variant analysis in GWAS [18,19,20,21,22,23,27,65]. Different LMMs approaches have practical and unique benefits. For example, the key benefit of single-locus modes is the power to deal with many markers, such as millions of markers. However, a single-locus-based method using a single locus at once fails to identify the correct genetic model of complex traits governed by various loci concurrently in GWAS [152]. The amendment of the multiple tests is another problem for the cut-off level of the significance test because the traditional Bonferroni correction is very stringent, resulting in numerous vital loci not exceeding the strict critical value of the significance test [24]. Importantly, multiple loci generally regulate complex traits, which cannot be tested using single-locus methods when each locus has a small effect [153]. Multi-locus LMMs are improved methods for GWAS as these methods do not need Bonferroni correction due to the multi-locus nature, and these methods showed more statistical power than singe locus methods [24,26,42,43,44]. These methods fail when the number of markers is numerous times higher than the sample size because of the limitation in memory allocation or computational complexity despite the usefulness of multi-locus LMMs in GWAS studies. For example, a multi-traits model named BOLT-LMM acquired more power over the present methods based on the conditions through its versatility prior to SNP effect size, depending on the exact genetic architecture and whether sample size are adequately substantial. This method is also sensitive to losing power when used to analyze large observed case-control data in low-incidence diseases. Data quality controlling is vital to elude false positives for correcting confounding factors. This method also has other limitations, such as being computationally slower than GRAMMAR-Gamma, not analyzing plant and animal data and considering only one random genetic effect in the model [51]. 

Recently, multi-traits association mapping has received more attention as these methods provide more power and in-depth knowledge for dissecting the genetic architecture of complex traits [154]. Many unmeasured aspects of the complicated biological network might be missed using single-trait analysis. Multi-traits analysis concurrently increases the power to grab these unmeasured prospects and identify more variants [71].

The statistical power of the multi-traits LMMs increases across traits by combining small genetic effects [57] and considering interrelated background distinction simplifying the decomposition of phenotypic variation into the diverse VC [63]. For example, GAMMA is developed for the generalized analysis of molecular variance for the mixed model, which is proficient in the instantaneous analysis of numerous phenotypes and controlling population structure [71]. SGL-LMM permits controlling confounding effects, consider the joint effects of multi-markers, and integrates biological group information as earlier knowledge [50]. Consequently, true genetic associations and better phenotypic prediction were possible by SGL-LMM in cases of weak marker effects, powerful confounding effects, and complex situations underlying genetic models [50]. Moreover, the statistical challenge is the robust covariance matrix estimation for multi-traits analysis in statistical genetics to single-cell study. Advanced informative and scalable methods are needed to analyze the enormous plant phenotyping of thousands of individuals from structured crosses with hundreds-thousands of image-based phenotypes [7]. LiMMBo expands to achieve LMMs into the new era, permitting new composite genetic associations and a more instructive investigation of the fundamental biological consequences [85]. Nonetheless, the active use of these methods is fixed as they are computationally rigid for big sample sizes [154]. 

There are many benefits of using G × G and G × E interaction methods, including the detection of the genetic effects which are missed in the linear models, enhancing the GWAS power, and giving the fractional answer to the missing heritability. However, different G × G and G × E methods have limitations. For example, GxEMM has several limitations, such as it is very computationally intensive, considering Gaussian random effects, which reduce power; and it does not correct for G–E correlation, which is a familiar origin of bias in the fixed effect situation [155]. Additionally, GxEMM did not fit the full model, and random effect is not permitted at present [104]. Another G × E method named StructLMM is robust and powerful, but there are limitations. Firstly, this method did not consider the heritable properties of the environmental variables, which may produce spurious associations. Secondly, this method chose variants that strongly affect the phenotype to reduce the multiple testing problem. However, this screening technique is not good for genome-wide testing for G × E interaction [104]. Furthermore, this method is computationally intensive compared with traditional LMMs and does not support controlling relatedness. Moreover, variance components raise based on the size of the grid and are proportional to the exponentially with the number of random effects. Grid-LMM estimates are not precise for posterior inference of variance component sizes and are bound to Gaussian LMMs. Furthermore, the Grid-LMM method has not investigated LMMs with correlated random effects [106]. Thus, more novel methods are needed to analyze G × G and G × E effects. Moreover, data incorporation from various natures is required to understand the interaction between genetic and environmental factors completely. 

Gene expression data and GWAS are incorporated by the TWAS to discover gene-traits associations. TWAS methods are needed to overcome the limitations of the other methods. For example, most of the GWAS hits are in non-coding regions, and their biological explanation is unclear. Additionally, all information from GWAS proposes that complex traits are frequently controlled by many variants with minor or moderate effects. In contrast, a prominent part of risk variants with minor effects remain unidentified [114]. A TWAS method named LSMM was proposed to integrate the functional annotation data with GWAS, and results showed that the statistical power of this method increased compared with other methods in identifying risk variants and uncovering cell-type related annotation [114]. Another method, SMART, integrated multiple binary and continuous annotations to simplify the detection of trait-associated tissues for GWAS traits [115]. However, improved SNP annotation tools and a large sample size might help adapt diverse annotation incorporation methods in the coming days. Many LMMs-based software and tools have been developed, which are authoritative for dissecting complex traits, and these tools are available, such as freely available statistical R packages. Moreover, applying LMMs in the biological field has challenges; for example, understanding model output can be complicated for the variance components of random effects and the model selections for LMMs [30]. Furthermore, investigation of G × G and G × E effects is needed when incorporating the different omics data, including transcriptomic, metabolomics, proteomics, and genomics in GWAS, to depict the genetic architecture of variants for complex traits. These big omics data deliver an unlimited opportunity for biological knowledge, but incorporating the various omics information and environmental effects is challenging.

However, there are some major restrictions on the current LMMs approaches. Firstly, LMMs are computationally costly and require a long time to analyze big datasets compared with simple models. For example, the run time and memory needed by LMM models are the scale as the cube and square of the cohort size, respectively [57]. Secondly, the existing LMM methods fail to achieve maximum statistical power due to insignificant modeling premises concerning the genetic structure-based phenotypes [51]. Thirdly, the ordinary LMM indirectly assumes that all variants are causal and follow the independent Gaussian distributions with minor effects, but the reality is that complex traits do not always follow the normal distribution [156,157]. Moreover, LMMs are unsatisfactory when many uncommon variants are incorporated into the analysis, particularly when population stratifications are determined by current demographic alterations [158]. Furthermore, the excessive polygenicity of many traits can pose challenges when revealing fundamental biological processes, especially when thousands of variants individually have a slight effect on a trait [159,160]. Therefore, novel approaches are required to tackle polygenicity and assist in explaining the outcome of GWAS through mechanistic intuition [160].

## 4. Future Perspective 

**Figure 4:**
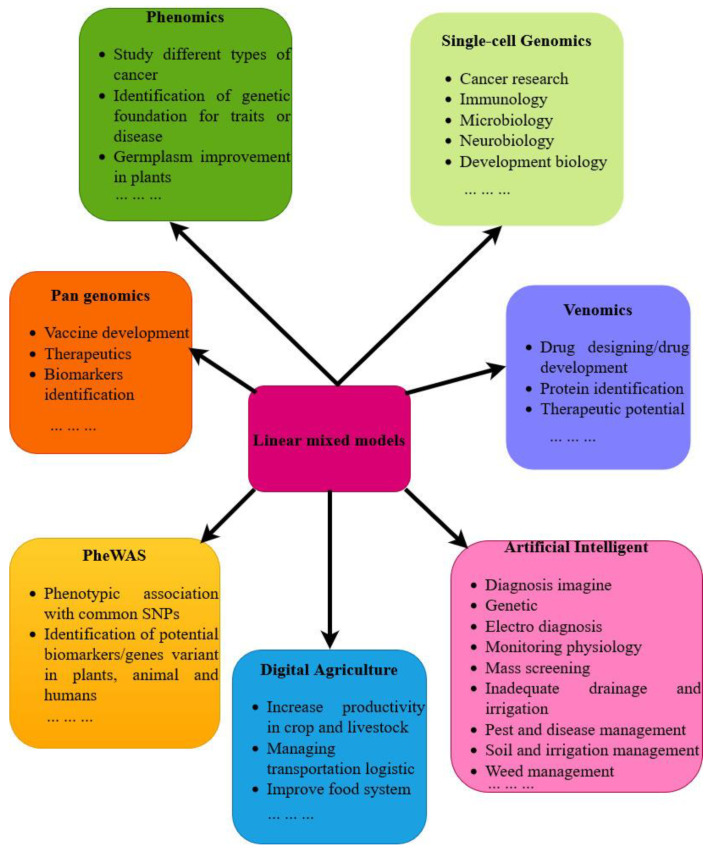
Linear mixed models (LMMs) could be used in the above potential fields currently developed.

The LMMs have been applied in most aspects of GWAS, including population stratification and relatedness, resulting in computational proficiency and increased statistical power in GWAS studies. However, genomes sequencing has rapidly increased due to the development of NGS, and genomic datasets are growing progressively [161]. This context incorporated the new research fields, including pan genomics, venomics, phenomics, single-cell genomics, and many others, with GWAS (Figure 4). Pan genomics compares the genetic content of diverse strains of similar species or genera, and there are few methods for the pan-genome data, but they give a biased estimate and enforce massive limitations in their models [161,162]. Another field, named phenomics, uses high-throughput data in genomics, which offers many facilities to acquire more worthy evidence than conventional procedures of plant phenotyping [163,164]. Furthermore, venomics is an interdisciplinary field investigating venoms, where different omics data, such as transcriptomics, genomics, and proteomics, are used [165]. Another new approach called pharmacometrics, incorporating different omics approaches has been developed to investigate vigorous molecular conditions for disease conditions and drug reactions [166]. 

Moreover, artificial intelligence (AI) is growing quickly due to its robust and stable application for resolving problems in conventional computing methods [167]. Furthermore, machine learning and other methods can obtain innovative understandings from meta-analyses of various datasets [164]. Likewise, deep learning is a widespread technique and is widely used in many fields as it can discover more complicated and nonlinear forms in big data [168,169]. Many modern technologies accelerate digital agriculture, including AI, robotics, remote sensing, and others, and these technologies support agriculturalists in acquiring complete, precise, crystal-clear crop and animal breeding products globally [170]. Although AI has received significant attention in agriculture and health research, the real application encounters problems. Additionally, difficulties and deficiencies, including methodologies to handle big data, storage, and computational bottleneck, should be overcome to successfully use these high technologies and the well-known digital revolution in agriculture [170]. Therefore, it is urgent and crucial to develop LMMs-based novel methods or software to analyze big omics data and dissect complex traits. 

## 5. Conclusions

This review introduced the available LMMs methods on GWAS, including single locus, multi-locus, multi-traits, TWAS, longitudinal GWAS, packages, and software in omics data. It provides a practical explanation and guides the reader to fundamental references that allow for an advanced methodological feature and better comprehension of GWAS. It also assists in finding appropriate LMM methods for dissecting complex traits in GWAS and further help to investigate these methods using diverse NGS and omics datasets. This review could guide both the new scholars and those desiring to update their knowledge in the field of GWAS by applying LMMs using the omics data. However, there is no unique and sophisticated software that users would like, including flexible and easy to use, combining different types of omics data, and which can handle big GWAS data analysis much faster than the existing methods. Necessary software and packages should be developed for analyzing big GWAS data sets and marker derivative kinship matrices. Overall, there is much scope to utilize the LMMs in diverse fields, including biostatistics, bioinformatics, and statistical genetics, which could be helpful for medical scientists, agriculturists, technologists, and data scientists to solve real-world problems.

## Data Availability

Not applicable.
